# Supplementary material for: Myriapod haemocyanin: the first three-dimensional reconstruction of Scolopendra subspinipes and preliminary structural analysis of S. viridicornis
Source: Open Biol. 2020 Apr 1;10(4):190258. doi: 10.1098/rsob.190258 (PMC7241075; doi:10.1098/rsob.190258)
Supplement: Figures S1 - S3 and Tables S1 - S2 [file rsob190258supp1.docx]

Myriapods Hemocyanin: The First 3D Reconstruction of *Scolopendra subspinipes and* Preliminary Structural Analysis of *S. viridicornis*

Riciluca KCT^1,2, §^, Borges AC^1, §^, Mello JFR^1^, Oliveira UC^2^, Serdan DC^1^, Florez-Ariza A^1^, Chaparro E^2,3^, Nishiyama-Junior MY^2^, Cassago A^1,^ Junqueira-de-Azevedo ILM^2^, van Heel M^1^, Silva Junior PI^2,3, †^, Portugal RV^1, ‡^

Supplementary figure

Figure S1 - Alignment of the amino acid sequences of Hemocyanin and Phenoloxidases from *Scolopendra subspinipes subspinipes* (Ssu) other myriapods, scorpions, arachnids and insects hemocyanins and phenoloxidases. Variations in blue scale indicate levels of sequence conservation. The symbol (-) represents gaps to improve the alignment. The species abbreviations are: Ssu1 - Ssu 8 - *Scolopendra subspinipes subspinipes*; SdeHc and SdePPO - *Scolopendra subspinipes dehaani*; SmaPPO - *Strigamia marítima*; PlaPPO - *Polyxenus lagurus*; PloPPO - *Panulirus longipes*; HamPPO - *Homarus americanos*; PlePPO - *Pacifastacus leniusculus*; FchiPPO *- Fenneropenaeu chinesis*; DmePPO - *Drosophila melanogaster*; MsexPPO - *Manduca sexta;* BmoPPO -*Bombyx mori* ; EpiHC *- Epiperipatus* sp.; EspHc1 - *Endeis spinosa*; LpoHc- *Limulus polyphemus*; CroHc -*Carcinoscorpius rotundicauda*; PimHc -*Pandinus imperator*; EcaHc- *Eurypelma californicum*; ScoHc - *Scutigera coleoptrata*; SpiHc - *Spirostreptus* sp.; AgiHc - *Archispirostreptus gigas*; PanHc - *Polysdesmus angustus*; SdeHc -*Scolopendra subspinipes dehaani*; HamHc - *Homarus americanos*; PleHc - *Pacifastacus leniusculus*; PinHc - *Panulirus interruptus*; CmaHc - *Cancer magister*; PvaHc - *Penaeus vannamei*; FchHc - *Fenneropenaeus chinensis*; TdoHc *- Thermobia domestic*a; PmaHc -*Perla marginata*; PamHc - *Periplaneta americana*

**Table S1– Table describing the animals used in Bayesian Tree**

| Acc. No | Species | Subphylum | Protein |
| --- | --- | --- | --- |
| ACM61982.1 | *Fenneropenaeus chinensis* | Crustacea | hemocyanin |
| CAB85965.1 | *Penaeus vannamei* | Crustacea | hemocyanin 1 |
| CAA57880.1 | *Penaeus vannamei* | Crustacea | hemocyanin |
| P80096.1 | *Panulirus interruptus* | Crustacea | hemocyanin c |
| AAW57891.1 | *Cancer magister* | Crustacea | hemocyanin 3 |
| AAW57892.1 | *Cancer magister* | Crustacea | hemocyanin 4 |
| AAW57893.1 | *Cancer magister* | Crustacea | hemocyanin 5 |
| AAA96966.2 | *Cancer magister* | Crustacea | hemocyanin 6 |
| P04254 | *Panulirus interruptus* | Crustacea | hemocyanin a |
| P10787 | *Panulirus interruptus* | Crustacea | hemocyanin b |
| CAB75960.1 | *Homarus americanus* | Crustacea | hemocyanin A |
| AAO47336.1 | *Pacifastacus leniusculus* | Crustacea | hemocyanin 2 |
| AAW57890.1 | *Cancer magister* | Crustacea | hemocyanin 2 |
| AAW57889.1 | *Cancer magister* | Crustacea | hemocyanin 1 |
| AAM81357.1 | *Pacifastacus leniusculus* | Crustacea | hemocyanin 1 |
| CAD87762.1 | *Perla marginata* | Hexapoda | hemocyanin 1 |
| CAR85701.1 | *Periplaneta americana* | Hexapoda | hemocyanin 1 |
| CAQ63321.1 | *Thermobia domestica* | Hexapoda | hemocyanin 1 |
| CAQ63322.1 | *Thermobia domestica* | Hexapoda | hemocyanin 2 |
| CAR85702.1 | *Periplaneta americana* | Hexapoda | hemocyanin 2 |
| CAD87763.1 | *Perla marginata* | Hexapoda | hemocyanin 2 |
| PanHC3 | *Polysdesmus angustus* | Myriapoda | hemocyanin 3 |
| CCC55877.1 | *Archispirostreptus gigas* | Myriapoda | hemocyanin 1 |
| CAC33894.1 | *Spirostreptus* sp. | Myriapoda | hemocyanin 1 |
| CCC55875.1 | *Polysdesmus angustus* | Myriapoda | hemocyanin 1 |
| Q8T116.1 | *Scutigera coleoptrata* | Myriapoda | hemocyanin X |
| Q8IFJ8.1 | *Scutigera coleoptrata* | Myriapoda | hemocyanin B |
| Ssu2 | *Scolopendra subspinipes subspinipes* | Myriapoda | x |
| SMH67861.2 | *Scolopendra subspinipes dehaani* | Myriapoda | hemocyanin B |
| Ssu1 | *Scolopendra subspinipes subspinipes* | Myriapoda | x |
| SMH67860.1 | *Scolopendra subspinipes dehaani* | Myriapoda | hemocyanin A |
| Q95P08.1 | *Scutigera coleoptrata* | Myriapoda | hemocyanin A |
| Q8T11S.1 | *Scutigera coleoptrata* | Myriapoda | hemocyanin C |
| Q95P07.1 | *Scutigera coleoptrata* | Myriapoda | hemocyanin D |
| CCC55876.1 | *Polysdesmus angustus* | Myriapoda | hemocyanin 2 |
| CCA94913.1 | *Endeis spinosa* | Chelicerata | Hemocyanin 1 |
| CAJ91100.1 | *Limulus polyphemus* | Chelicerata | Hemocyanin VI |
| AAZ40341.1 | *Carcinoscorpius rotundicauda* | Chelicerata | Hemocyanin VI |
| AAZ40340.1 | *Carcinoscorpius rotundicauda* | Chelicerata | Hemocyanin V |
| CAZ66714.1 | *Pandinus imperator* | Chelicerata | Hemocyanin 3C |
| Q9NFL6.3 | *Eurypelma californicum* | Chelicerata | hemocyanin C |
| CAZ66719.1 | *Pandinus imperator* | Chelicerata | Hemocyanin 5B |
| Q9NFH9.3 | *Eurypelma californicum* | Chelicerata | hemocyanin B |
| AAZ40336.1 | *Carcinoscorpius rotundicauda* | Chelicerata | Hemocyanin II |
| CAJ91097.1 | *Limulus polyphemus* | Chelicerata | Hemocyanin II |
| CAZ66715.1 | *Pandinus imperator* | Chelicerata | Hemocyanin 3B |
| CAZ66712.1 | *Pandinus imperator* | Chelicerata | Hemocyanin 3A |
| P14750.3 | *Eurypelma californicum* | Chelicerata | hemocyanin A |
| CAZ66718.1 | *Pandinus imperator* | Chelicerata | Hemocyanin 4 |
| Q9NFL4.3 | *Eurypelma californicum* | Chelicerata | hemocyanin G |
| CAZ66716.1 | *Pandinus imperator* | Chelicerata | Hemocyanin 6 |
| P02242.3 | *Eurypelma californicum* | Chelicerata | hemocyanin E |
| P02241.3 | *Eurypelma californicum* | Chelicerata | hemocyanin D |
| CAZ66717.1 | *Pandinus imperator* | Chelicerata | Hemocyanin 5A |
| Q9NFL5.3 | *Eurypelma californicum* | Chelicerata | hemocyanin F |
| CAZ66713.1 | *Pandinus imperator* | Chelicerata | Hemocyanin 2 |
| CAJ91099.1 | *Limulus polyphemus* | Chelicerata | Hemocyanin IV |
| AAZ40339.1 | *Carcinoscorpius rotundicauda* | Chelicerata | Hemocyanin IV |
| AAZ40338.1 | *Carcinoscorpius rotundicauda* | Chelicerata | Hemocyanin IIIb |
| CCA94914.1 | *Limulus polyphemus* | Chelicerata | Hemocyanin IIIb |
| AAZ40335.1 | *Carcinoscorpius rotundicauda* | Chelicerata | Hemocyanin I |
| AAZ40337.1 | *Carcinoscorpius rotundicauda* | Chelicerata | Hemocyanin IIIa |
| CAJ91098.1 | *Limulus polyphemus* | Chelicerata | Hemocyanin IIIa |
| CAD12808.1 | *Epiperipatus* sp. | Onychophora | Hemocyanin |
| CCC55880.1 | *Polyxenus lagurus* | Myriapoda | Prophenoloxidase |
| SMH678664.1 | *Scolopendra subspinipes dehaani* | Myriapoda | Prophenoloxidase |
| Ssu3 | *Scolopendra subspinipes subspinipes* | Myriapoda | x |
| SmaPPO | *Strigamia maritima* | Myriapoda | Prophenoloxidase |
| SMH678662.3 | *Scolopendra subspinipes dehaani* | Myriapoda | Prophenoloxidase |
| Ssu7 | *Scolopendra subspinipes subspinipes* | Myriapoda | x |
| Ssu4 | *Scolopendra subspinipes subspinipes* | Myriapoda | x |
| Ssu5 | *Scolopendra subspinipes subspinipes* | Myriapoda | x |
| Ssu6 | *Scolopendra subspinipes subspinipes* | Myriapoda | x |
| Ssu8 | *Scolopendra subspinipes subspinipes* | Myriapoda | x |
| SMH678663.2 | *Scolopendra subspinipes dehaani* | Myriapoda | Prophenoloxidase |
| NP_610443.1 | *Drosophila melanogaster* | Hexapoda | Prophenoloxidase 2 |
| NP_524760.1 | *Drosophila melanogaster* | Hexapoda | Prophenoloxidase 3 |
| NP_001037335.1 | *Bombyx mori* | Hexapoda | Prophenoloxidase 1 |
| 3HHS-B | *Manduca sexta* | Hexapoda | Prophenoloxidase 1 |
| 3HHS-A | *Manduca sexta* | Hexapoda | Prophenoloxidase 2 |
| NP_001037534.1 | *Bombyx mori* | Hexapoda | Prophenoloxidase 2 |
| BAA08272.1 | *Drosophila melanogaster* | Hexapoda | Prophenoloxidase A1 |
| AGI42859.1 | *Fenneropenaeu chinensis* | Crustacea | Prophenoloxidase 2 |
| ADD70249.1 | *Panulirus longipes* | Crustacea | Prophenoloxidase |
| AAT73697.1 | *Homarus americanus* | Crustacea | Prophenoloxidase |
| CAA58471.1 | *Pacifastacus leniusculus* | Crustacea | Prophenoloxidase |
| AGI42860.1 | *Fenneropenaeu chinensis* | Crustacea | Prophenoloxidase 3 |

**Table S2 – Summary Statistic Beast**

| **Summary Statistic** |  | **run1** | **run2** | **run3** | **run4** |
| --- | --- | --- | --- | --- | --- |
| **mean** |  | -70.065,70 | -70.066,33 | -70.066,27 | -70.065,19 |
| **stderr of mean** |  | 0.282 | 0.3495 | 0.27 | 0.2697 |
| **stdev** |  | 10,55 | 10,45 | 10,40 | 10,25 |
| **median** |  | -70.065,34 | -70.066,06 | -70.065,93 | -70.064,94 |
| **95% HPD Interval** |  | [-70.087,69 -70.046,34] | [-70.086,30 -70.045,65] | [-70.086,92 -70.045,99] | [-70.085,60 -70.045,32] |
| **auto-correlation time (ACT)** |  | 13.567,95 | 21.392,63 | 12.851,05 | 13.210,79 |
| **effective sample size (ESS)** |  | 1.398,22 | 893,49 | 1.482,76 | 1.445,03 |

ScoHcA/1-638 416 HTYWEVDNYELGKGFDYTRK-TTATVKVRHLQHEDYHYEIDIDNNAGKAKKAVFRIFLAP
TRINITY_DN5415/ 385 KTFWEDDVLTVGTGFTFTGPSATAKVNIRHLEHEEFSYNIQVMNNGGENKKAVFRIFLAP
consensus 421 .*.** * .* ** .* . ** * .*** **.. * * . **.* ***********

ScoHcA/1-638 475 KYNEKGELFPVNEQRQLLVELDKFVATLEPGHNVIERQSKESSVTMSKDHVFGEIRNLAD
TRINITY_DN5415/ 445 KYDEKGHEYDFNEQRQSMIELDKFVKELTPGKNVVIRKSSESSVTQKHEKIYANPKERQQ
consensus 481 ** *** . ***** ..****** * **.**. * * ***** ...... .

ScoHcA/1-638 535 DHQCSCGWPDYLLLPKGKYEGMTYQLFVVATDYEEDHVEDAGEECQCRDSMSYCGSVEHK
TRINITY_DN5415/ 505 NDHCSCGWPDNLLVPRGSYEGTEFQVFVVVTNYKEDYVP-SDESCHCGDGRSYCGILFGN
consensus 541 ******* **.*.* *** .*.*** * * ** * . * * * * **** .

Figure S2– Alignment of the amino acid sequences of subunit of hemocyanin ScoHcA (*Scutigera coleoptrata*) and Ssu2 (*Scolopendra subspinipes subspinipes*). The red square showed the similarity


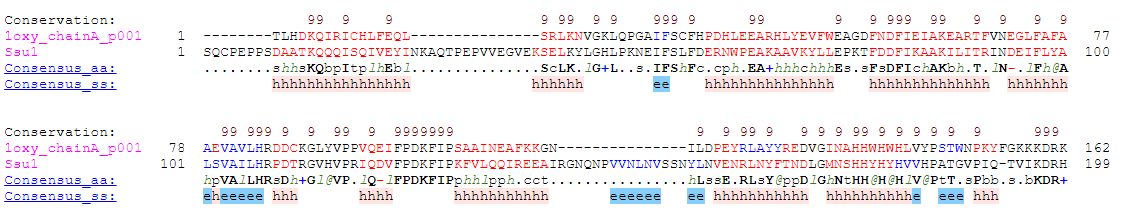


Figure S3 - Alignment of the amino acid sequences of subunit II of hemocyanin 1OXY (*Limulus polyphemus*) and Ssu1 (*Scolopendra subspinipes subspinipes*). The red square showed the gap that represents the loop in the structure.
